# The utility of joinpoint regression for estimating population parameters given changes in population structure

**DOI:** 10.1016/j.heliyon.2019.e02515

**Published:** 2019-11-19

**Authors:** Daniel Gillis, Brandon P.M. Edwards

**Affiliations:** a University of Guelph, School of Computer Science, 50 Stone Road East, Guelph, ON N1G2W1 Canada; b University of Guelph, Department of Mathematics & Statistics, 50 Stone Road East, Guelph, ON N1G2W1 Canada

**Keywords:** Applied mathematics, Computational mathematics, Joinpoint, Surplus production model, Simulation study, Population parameter

## Abstract

The method of joinpoint regression has been used in numerous domains to assess changes in time series data, including such things as cancer mortality rates, motor vehicle collision mortalities, and disease risk. To help improve estimation of population parameters for use in ecological risk assessment and management, we present a simulation and analysis to describe the utility of this method for the ecological domain. We demonstrate how joinpoint regression can accurately identify if the population structure changes based on time series of abundance, as well as identify when this change occurs. In addition, we compare and contrast population parameter estimates derived through joinpoint and surplus production methods to those derived from standard surplus production methods alone. When considering a change point at 32 years (out of a 64 year simulation), the joinpoint regression model was able, on average, to estimate a joinpoint time of 32.31 years with a variance of 6.82 and 95% confidence interval for the mean relative bias of (0.0085, 0.0112). The model was able to consistently estimate population parameters, with variance of these estimations decreasing as the change in these population parameters increased. We conclude that joinpoint regression be added to the list of methods employed by those who assess ecological risk to allow for a more accurate and complete understanding of population dynamics.

## 1 Introduction

As the global population approaches an estimated 9 billion people by 2030 (Department of Economic & Social Affairs: Population Division, 2017), the need to sustainably manage populations of species that provide food or economic value to communities becomes paramount. This is particularly important to remote Indigenous populations that rely on country-food for subsistence, wellbeing, tradition, and culture. Unfortunately, the literature is full of examples where over-harvesting, loss of breeding-habitat, or other anthropogenic activities have negatively affected the sustainability of a wild animal population (see, for example (Flockhart et al., 2014; Poudyal et al., 2009; Rushing et al., 2016)). Two recent and well known Canadian examples include the decline of the northern caribou (*Rangifer tarandus groenlandicus/Rangifer tarandus granti*), and the collapse of the Atlantic cod (*Gadus morhua*) industry of Newfoundland and Labrador. In the former case, caribou numbers have fallen significantly in the last 20 years, with current population estimates 70% lower than observed in the late nineties (Parlee et al., 2018). Recent research has indicated that the population collapse is likely attributed to the effect of mining operations in Northern Canada (Parlee et al., 2018). In the latter case, increased harvesting led to the collapse of the Atlantic cod population and industry in the early 1990s (Hutchings and Myers, 1994; Myers et al., 1996). After almost 30 years, the population has not returned to its former levels (Pederson et al., 2017). In both cases, the effects have been profound and widespread, negatively affecting employment, local economies, and the various dimensions of health and wellbeing (Gien, 2000; Prowse et al., 2009).

Risk assessment models, such as the surplus production model, the stage-structured model, or the agent-based model, are often used by managers and decision makers to assess and evaluate the status of animal populations (Hilborn and Walters, 2001; Rose et al., 2017). While varying in complexity, the models provide managers with estimates of population parameters which can be used to understand the current state of the population and to project the effect of various management decisions on the sustainability of the population in question.

### 1.1 Surplus production models

The surplus production models, also known as biomass dynamic models, is a simple yet effective model used in fishery science for stock assessment (Hilborn and Walters, 2001). In surplus production models, the population's biomass *B* at time *t* given population growth rate *r*, carrying capacity *K*, and harvest *C* is given by:

(1) $$B_{t}=B_{t-1}+rB_{t-1}\left(1-\frac{B_{t-1}}{K}\right)-C_{t-1}$$

where the harvest *C* at time *t* given catchability coefficient *q* and harvest efforts *E* is given by:

(2) $$C_{t}=qB_{t}E_{t}$$

In most cases, these parameters are assumed to be constant across the time series, or drawn from a particular distribution (Hilborn and Walters, 2001). Using estimates of these population parameters allows managers to estimate the population's maximum sustainable yield for a particular period of time (Polacheck et al., 1993). This information can be used to evaluate management decisions under the assumption that the estimates are estimated well, and that there will be no change to them in the foreseeable future.

However, standard surplus production models and other risk assessment models fail to account for sudden changes in population dynamics that result from, for example, anthropogenic activities. Changes to migration pathways, loss of breeding grounds, or other issues associated with industrial development, climate change, or pollution (for example) could suddenly alter the dynamics and long-term stability of a population. Failure to consider changes to the dynamics of the population during the modelling process might lead to estimates of population parameters that fail to provide realistic projections of management decisions. For animal populations already suffering from the effects of anthropogenic activities, management actions based on poorly assessed data could be disastrous. As such, there is a need to explore risk models that allow for changes in population structure within an observed time series of data, as this could improve our understanding of the dynamics of a population and improve our ability to manage it effectively and sustainably (Sprent, 1961).

### 1.2 Joinpoint regression

One method that has been identified as a valuable tool for making inferences about changes in trends over time is joinpoint regression (see for example Barrio et al. (2015); Lopez-Campos et al. (2014); Bosetti et al. (2011); Long et al. (2014); Yu et al. (2009)). It has been used, for example, to accurately estimate changes in rates of driver deaths after the implementation of new traffic regulations (Barrio et al., 2015), to estimate changing trends in cancer incidence in countries such as Canada, United Kingdom, Japan, and Italy (among many other countries) (Jiang et al., 2010; Qiu et al., 2009; Crispo et al., 2013; Wilson et al., 2017), and to analyze changes in suicide rates in Denmark (Dyvesether et al., 2018). These studies, all of which assess changes in time series data that happen following various interventions or changes in external conditions, suggest the potential utility of joinpoint regression to identify and evaluate when/if changes in population parameters occur. To our knowledge, however, joinpoint regression has not been used in this manner during the risk assessment process of populations of animals.

Joinpoint regression, also known as change point regression or segmented regression, assumes that data can be divided into subsets - each with their own unique linear trend. For example, if we were to analyze a time series with two different trends, we might find that when $t<t^{⁎}$, the model has a particular intercept and slope, and when $t\geq t^{⁎}$, the model would have a different intercept and slope. In this instance, $t^{⁎}$ represents the joinpoint; a point in time when the population parameters change. In a population setting, joinpoint regression could be used to improve models in the case where anthropogenic activities, for example, move a species from a period of stability to a period of decline.

In general, given data vector $y=(y_{1},y_{2},\ldots ,y_{n})$ with covariate vector $x=(x_{1},x_{2},\ldots ,x_{n})$, and joinpoint vector $\tau =(\tau_{1},\ldots ,\tau_{k})$, a joinpoint model is described as:

(3) $$y_{i}=\left\{ \begin{array}{cc} \beta_{0}+\beta_{1}x_{i}+\epsilon_{i},\; & x_{i}<\tau_{1}\\ \beta_{0}+\beta_{1}x_{i}+\delta_{1}(x_{i}-\tau_{1})+\epsilon_{i},\; & \tau_{1}\leq x_{i}<\tau_{2}\\ \vdots \; & \vdots \\ \beta_{0}+\beta_{1}x_{i}+\delta_{k-1}(x_{i}-\tau_{k-1})+\epsilon_{i},\; & \tau_{k-1}\leq x_{i}<\tau_{k}\\ \beta_{0}+\beta_{1}x_{i}+\delta_{k}(x_{i}-\tau_{k})+\epsilon_{i},\; & \tau_{k}\leq x_{i} \end{array}\right.,$$

where $\beta_{0},\beta_{1},\delta_{1},\ldots \delta_{k}$ are regression coefficients (with $\delta_{1},\ldots \delta_{k}$ being slope differences given time period *τ*) and $\epsilon_{i}∼N(0,\sigma^{2})$. That is, the joinpoint model assumes linearity, and errors $\epsilon_{i}$ are independent and normally distributed (Sprent, 1961; Hinkley, 1971; Jiang et al., 2010).

This paper will explore the use of joinpoint regression analysis in the area of ecological risk assessment. More specifically, the goal of this paper is to determine the effectiveness of joinpoint regression to improve the estimates of population parameters derived from the standard surplus production model. To achieve this goal it becomes necessary to satisfy the following objectives: 1) determine if joinpoint regression can be used to identify if and when population structure changes occur based on time series of abundance, and 2) compare and contrast population parameter estimates derived through joinpoint and surplus production methods to those derived from standard surplus production methods alone.

## 2 Methods

To evaluate the effectiveness of joinpoint regression to improve the estimation of population parameters over a range of situations, a standard surplus production model as in Equation (1) was developed to allow for a change in the growth rate (*r*), and the catchability coefficient (*q*), in year 32 of a 64 year time series. Specifically, Equation (1) was reformulated in terms of a general joinpoint equation as in Equation (3), and data were simulated from the standard surplus production model

(4) $$B_{t}=\left\{ \begin{array}{cc} B_{t-1}+r_{1}B_{t-1}\left(1-\frac{B_{t-1}}{K}\right)-C_{t-1},\; & t<32\\ B_{t-1}+r_{2}B_{t-1}\left(1-\frac{B_{t-1}}{K}\right)-C_{t-1},\; & t\geq 32 \end{array}\right.,$$

where *B*, *r* (separated into $r_{1}$ and $r_{2}$), *K*, and *C* are as described in Equation (1), and *t* is the discrete time step in years. Harvest *C* as in Equation (2) was also reformulated in terms of a general joinpoint equation as in Equation (3) to arrive at

(5) $$C_{t}=\left\{ \begin{array}{cc} q_{1}B_{t}E_{t}e^{\epsilon_{t}},\; & t<32\\ q_{2}B_{t}E_{t}e^{\epsilon_{t}},\; & t\geq 32 \end{array}\right.,$$

where $E_{t}$ is the total yearly effort in year *t* measured in metres of net, and sampled from a Poisson distribution with mean parameter λ=10,000, $\epsilon_{t}∼N(0,10^{-1})$ is a random normal variate, and $q_{1}$ and $q_{2}$ are the catchability coefficients for period 1 and period 2, respectively.

For each simulation, $r_{1}$, $r_{2}$, $q_{1}$, and $q_{2}$ were randomly sampled as follows: $r_{1}$ was randomly drawn from the set {0.55, 0.60, 0.65, 0.70, 0.75, 0.80, 0.85, 0.90}, $r_{2}$ was randomly drawn from the set {0.20, 0.25, 0.30, 0.35, 0.40, 0.45, 0.50, 0.55}, $q_{1}$ was randomly drawn from the set {$1.0\times 10^{-5}$, $1.5\times 10^{-5}$, $2.0\times 10^{-5}$, $2.5\times 10^{-5}$}, and $q_{2}$ was randomly drawn from the set {$2.0\times 10^{-5}$, $2.5\times 10^{-5}$, $3.0\times 10^{-5}$}. The sets were created to ensure $r_{1}\geq r_{2}$, and to allow for a range of changes to growth rate, and to the catchability coefficient (identified by $\delta_{r}=r_{1}-r_{2}$, and $\delta_{q}=q_{1}-q_{2}$, respectively) at time t=32.

**Figure 1:**
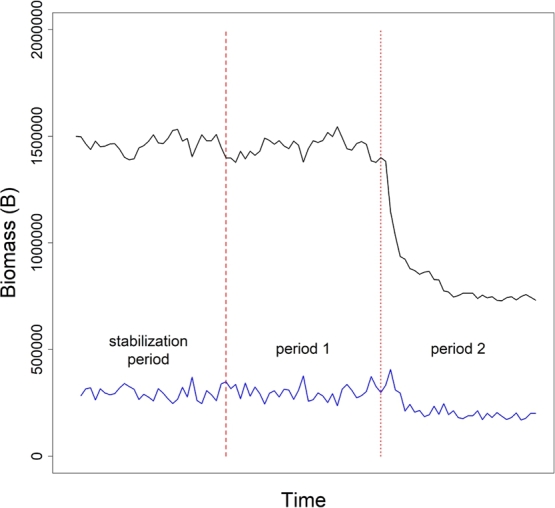
Simulated time series of biomass (black line) and catch (blue line) data by year. Data were generated assuming *B*_0_ = 1,500,000, and *K* = 2,000,000. For each simulation, 32 years of data (*stabilization period*, to the left of the dashed red line) were generated to ensure the simulated population was stable. Following this, data were generated assuming growth rate *r*_1_ and catchability coefficient *q*_1_ up to year 64 (*period 1*, between the dashed and dotted red lines). Finally, the population structure was updated to allow a growth rate of *r*_2_ and catchability coefficient of *q*_2_ (*period 2*, to the right of the dotted red line).

Once the growth rates and catchability coefficients were selected, $B_{0}$ was set to 1,500,000, and *K* to 2,000,000. Equations (4) and (5) were updated with $r_{1}$, $q_{1}$, $B_{0}$, and *K*, and allowed to run for 32 years to allow the simulated population to stabilize (see *stabilization period* in Fig. 1). At the end of the 32 year stabilization period, *t* was reset to 0, and $B_{0}$ was set to $B_{32}$ (see *period 1* in Fig. 1). The simulation was allowed to run for another 32 years, at which $r_{1}$ and $q_{1}$ were updated to $r_{2}$ and $q_{2}$. Finally, the simulation was allowed to run for another 32 years. Time series of both *B* and *C* were extracted and processed for each of the following three scenarios:

1. **Scenario** 1 **(*joinpoint ignored*)**: the 64 year time series catch data were analyzed using a standard surplus production model, ignoring the joinpoint at time t=32,
2. **Scenario** 2 **(*joinpoint estimated*)**: the 64 year time series catch data were analyzed using the surplus production model described by Equations (4) and (5), using an estimated joinpoint, and
3. **Scenario** 3 **(*joinpoint known*)**: the 64 year time series catch data were analyzed using the surplus production model described by Equations (4) and (5), assuming that the true joinpoint was known.

To estimate the growth rate(s) and catchability coefficient(s), Equation (4) was multiplied by $\frac{1}{K}$ and then $B_{t}$ was expressed as a proportion of *K* (i.e. $P_{t}=\frac{B_{t}}{K}$), and Equation (5) was re-expressed in terms of catch per unit effort $I_{t}$ (i.e. $I_{t}=\frac{C_{t}}{E_{t}}$):

(6) $$P_{t}=\left\{ \begin{array}{cc} P_{t-1}+r_{1}P_{t-1}\left(1-P_{t-1}\right)-\frac{C_{t-1}}{K},\; & t<32\\ P_{t-1}+r_{2}P_{t-1}\left(1-P_{t-1}\right)-\frac{C_{t-1}}{K},\; & t\geq 32 \end{array}\right.,$$

(7) $$I_{t}=\left\{ \begin{array}{cc} q_{1}P_{t}K,\; & t<32\\ q_{2}P_{t}K,\; & t\geq 32 \end{array}\right..$$

The model was implemented using the JAGS software (see JAGS Code in Supplementary Files), and evaluated using the *rjags* and *coda* packages in R (see R Code in Supplementary Files) (Plummer, 2015; R Core Team, 2014; Plummer et al., 2006). Bayesian priors were assigned assuming $P_{t}$ and $I_{t}$ had log normal distributions with means as described for each scenario below. To ensure reasonable samples for $P_{t}$, the distributions were truncated above and below. Finally, distributions for $P_{t}$ and $I_{t}$ were assigned variances *σ* and *τ*, respectively. Both $\sigma^{-1}$ and $\tau^{-1}$ were assigned Gamma hyper priors.

Simulated catch and harvest data were analyzed for each of the scenarios in the following ways:

• **Scenario** 1 **(*joinpoint ignored*)**: Equations (6) and (7) were updated to include a single growth rate, and a single catchability coefficient. That is, $r_{1}$ and $r_{2}$ were replaced with *r*, and $q_{1}$ and $q_{2}$ were replaced with *q*. Further, log normal priors for $P_{t}$ and $I_{t}$ were assigned means of $P_{t-1}+rP_{t-1}\left(1-P_{t-1}\right)-\frac{C_{t-1}}{K}$, and $qP_{t}K$.
• **Scenario** 2 **(*joinpoint estimated*)**: Equations (6) and (7) were updated for scenario 2 to reflect the case where the joinpoint was estimated. Specifically, letting $t^{⁎}$ represent the estimated joinpoint, we replaced $r_{1}$ and $q_{1}$ with $r_{1}^{⁎}$ and $q_{1}^{⁎}$ when $t<t^{⁎}$, and $r_{2}$ with $r_{2}^{⁎}$ and $q_{2}$ with $q_{2}^{⁎}$ when $t\geq t^{⁎}$. Log normal priors were assigned means of $P_{t-1}+r_{i}^{⁎}P_{t-1}\left(1-P_{t-1}\right)-\frac{C_{t-1}}{K}$, and $q_{i}^{⁎}P_{t}K$, respectively (where i=1 if $t<t^{⁎}$, and i=2 if $t\geq t^{⁎}$). To estimate the time of the joinpoint required for scenario 2, a simple linear model (i.e. $I_{t}=\beta_{0}+\beta_{1}t$) was created, and used with the *segmented* package in R (R Core Team, 2014; Muggeo, 2003, Muggeo, 2008). Although the joinpoint was known to occur at time t=32, the *segmented* function was parameterized and initialized to allow for two joinpoints. This was repeated five times, with initialized joinpoint values of (16, 48), (18, 46), (20, 44), (22, 42), and (24, 40). The decision to use two joinpoints would account for changes to population dynamics that might present themselves over the course of several years, instead of instantaneously. In this way, the *segmented* function might identify three distinct periods within the population structure, instead of simply pre and post joinpoint. For example, there could be a stable period of abundance prior to the joinpoint, followed by a period where abundance decreases or increases to a new period of stability. Estimated joinpoints $t_{1i}^{⁎}$ and $t_{2i}^{⁎}$, where $t_{1i}^{⁎}\leq t_{2i}^{⁎}$, were saved for each initialization set (i=1,…,5). The rounded average of the smaller joinpoint from each of the initialization sets was used to parameterize the JAGS model described previously. That is, $t^{⁎}$ was set $\frac{1}{5}\sum_{i=1}^{5}t_{1i}^{⁎}$, rounded to the nearest integer value.
• **Scenario** 3 **(*joinpoint known*)**: Analysis proceeded using Equations (6) and (7) without changes. Log normal priors were assigned means of $P_{t-1}+r_{i}^{⁎}P_{t-1}\left(1-P_{t-1}\right)-\frac{C_{t-1}}{K}$, and $q_{i}^{⁎}P_{t}K$, respectively (where i=1 if t<32, and i=2 if t≥32).

**Figure 2:**
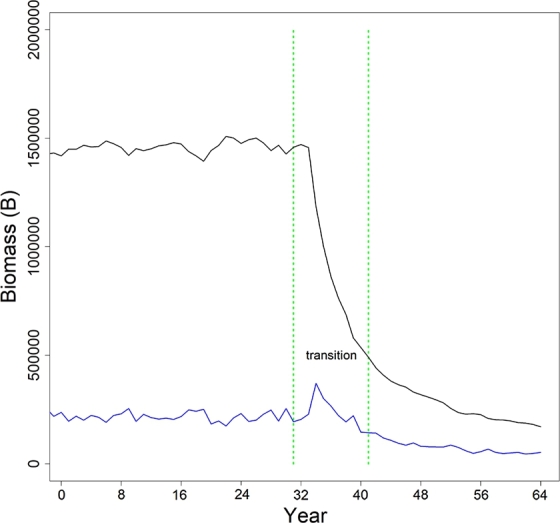
Estimated joinpoints (green line) given simulated time series of abundance (black line) and catch (blue line) data by year. Catch data were simulated assuming *B*_0_ = 1,500,000, and *K* = 2,000,000 for 64 years. At time *t* = 32 years, the population dynamics were adjusted to allow for a change in growth rate and catchability coefficient. The transition period (identified as the period between the two estimated joinpoints) represents the estimated length of time for the population to restabilize following a change in the dynamics.

In the cases where the joinpoint analyses would not converge to a solution, the simulation was rejected and new draws for $r_{1}$, $r_{2}$, $q_{1}$, and $q_{2}$ were made. In total, 13,513 simulations were conducted. Output from each of the three scenarios were summarized using the *coda* package in R (R Core Team, 2014; Plummer et al., 2006). Results were analyzed to evaluate the ability of the joinpoint regression to i) estimate the true joinpoint time $t^{⁎}$, ii) estimate the true joinpoint versus the change in growth rate between periods 1 and 2 (i.e. $\delta_{r}=\left|r_{1}-r_{2}\right|$), iii) estimate the true joinpoint versus the change in catchability coefficients between periods 1 and 2 (i.e. $\delta_{q}=\left|q_{1}-q_{2}\right|$), iv) identify the length of the transition period during which a stable population appeared following the joinpoint (i.e. $\mathrm{abs}\left(t_{1}^{⁎}-t_{2}^{⁎}\right)$, where $t_{1}^{⁎}$ has been previously defined, and $t_{2}^{⁎}=\frac{1}{5}\sum_{i=1}^{5}t_{2i}^{⁎}$) - see Fig. 2 - and, v) improve the estimates of growth rate and catchability compared to standard methods (i.e. when joinpoints are ignored), or when the joinpoint is known *a priori*.

All simulations and analyses were completed using R: A Language and Environment for Statistical Computing (R Core Team, 2014) with a 13” MacBook Pro with a 2.7 GHz Intel Core i5 processor, and 16 GB 1867 MHz DDR3 RAM.

## 3 Results

**Figure 3:**
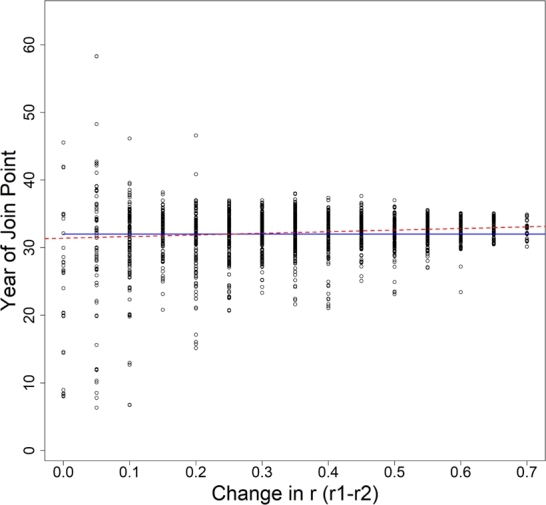
Estimated year of joinpoints (black dots) for each simulation versus the change in growth rate (pre and post joinpoint). The blue line represents the true joinpoint value of *t* = 32 years. The red dashed line represents a simple linear regression of year of joinpoint versus change in growth rate.

Ignoring the randomly selected growth rates and catchability coefficients, the average estimated joinpoints $\overset{ˆ}{t_{1}^{⁎}}$ and $\overset{ˆ}{t_{2}^{⁎}}$ were 32.31 and 39.50, with variances of 6.82 and 14.05, respectively. The mean relative bias of $\overset{ˆ}{t_{1}^{⁎}}$ across all simulations was slightly positive. Specifically, the 95% confidence interval for the mean relative bias was (0.0085, 0.0112). Since $\overset{ˆ}{t_{2}^{⁎}}$ is an artifact of the analytical method and not reflective of a known value, relative bias can not be calculated. The average estimated joinpoint is consistent regardless of the magnitude of $\delta_{r}$, however, the variance of the estimated values decreases as $\delta_{r}$ increases (see Fig. 3).

For low values of $\delta_{r}$ (e.g. $\delta_{r}<0.10$), variance estimates are approximately 5 times that of those seen for $\delta_{r}\geq 0.10$. There is a slight upward trend in mean estimated joinpoint with increasing magnitude of $\delta_{q}$. The variance of the estimated joinpoints appears to be stable except in the case of larger $\delta_{q}$, where the variation appears to double. However, there are fewer simulations with high $\delta_{q}$, so this uptick may itself be a deviation from the norm. On average the transition period is approximately 7 years; a pattern which is consistent regardless of the magnitude of $\delta_{r}$ and $\delta_{q}$. That is, following a change in growth rate and/or catchability coefficient, it takes on average 7 years for the population to restabilize. However, the transition period ranges in length from as little as 1.16 years, to as much as 50.85 years. Shorter transition periods are associated with higher values of $\delta_{r}$ and higher values of $\delta_{q}$.

**Figure 4:**
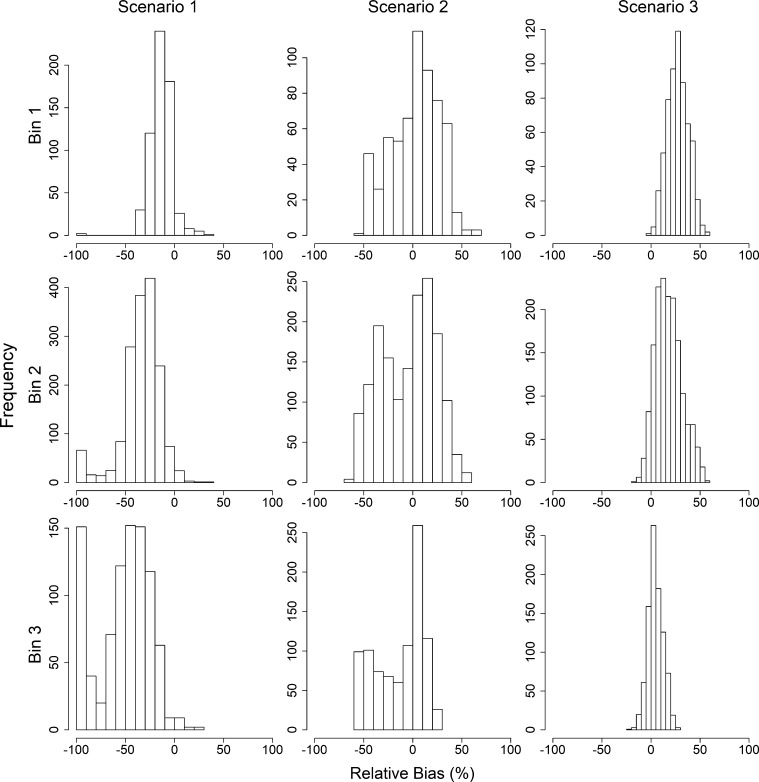
Relative bias (%) of growth rate *r*_1_ given *δ*_*r*_ = *r*_1_ − *r*_2_, where *r*_1_ is the growth rate prior to the joinpoint, and *r*_2_ is the growth rate following the joinpoint, by scenario. Delta growth bins 1–3 (rows) include experiments where *δ*_*r*_ ≤ 0.20, 0.20 < *δ*_*r*_ ≤ 0.45, and *δ*_*r*_ > 0.45, respectively. Scenario 1–3 (columns) indicate analysis assuming no joinpoint, using the estimated joinpoint, or using the true joinpoint, respectively.

Distributions of the relative bias of the growth rate parameter $r_{1}$ are provided in Fig. 4 for each scenario. For simplicity, the relative bias estimates are summarized given the magnitude of the change in growth before and after the joinpoint. Specifically, they are grouped based on the $\delta_{r}$ (see caption of Fig. 4 for binning).

The mean relative bias for $r_{1}$ is negative for all binning situations when the experiments were analyzed assuming no change to the population dynamics structure. That is, when there is no change in the growth rate or the catchability coefficient (i.e. Scenario 1), $r_{1}$ is significantly underestimated, with average relative bias estimates for the largest $\delta_{r}$ of −51%. The magnitude of the relative bias increases as $\delta_{r}$ increases. Further, the relative bias also increases across scenarios within the $\delta_{r}$ bins. That is, for all $\delta_{r}$ bins, the relative bias is largest when using the true joinpoint. This is followed by the experiments using an estimated joinpoint, and finally by the experiments that ignored the joinpoint. For all $\delta_{r}$ bins, the average relative bias was positive, but decreased as $\delta_{r}$ increased. Average relative bias estimates can be found in Table 1.

**Table 1:** Mean relative bias of growth rate *r*_1_ (and standard deviation) given *δ*_*r*_ = *r*_1_ − *r*_2_ bins, where *r*_1_ is the growth rate prior to the joinpoint, and *r*_2_ is the growth rate following the joinpoint, by scenario. Scenario 1–3 indicate analysis assuming no joinpoint, using the estimated joinpoint, or using the true joinpoint, respectively.

Bin	Scenario 1	Scenario 2	Scenario 3
*δ*_*r*_ ∈ [0.00,0.20]	−13.73 (0.46)	2.28 (0.99)	27.36 (0.43)
*δ*_*r*_ ∈ (0.20,0.45]	−33.80 (0.49)	−4.96 (0.69)	18.06 (0.33)
*δ*_*r*_ ∈ (0.45,0.70]	−51.68 (0.89)	−13.11 (0.79)	4.75 (0.25)

As outlined in Table 1, the standard deviations of the mean relative bias increase as $\delta_{r}$ increases in Scenario 1, decreases as $\delta_{r}$ increases in Scenario 3, and fluctuates in Scenario 2.

**Figure 5:**
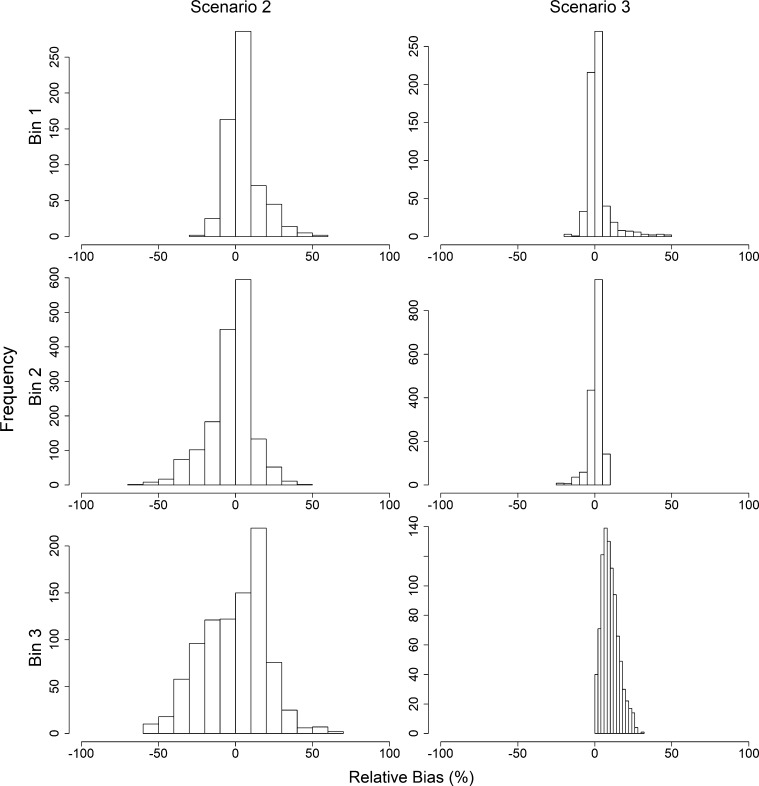
Relative bias (%) of growth rate *r*_2_ given *δ*_*r*_ = *r*_1_ − *r*_2_, where *r*_1_ is the growth rate prior to the joinpoint, and *r*_2_ is the growth rate following the joinpoint, by scenario. Delta growth bins 1–3 (rows) include experiments where *δ*_*r*_ ≤ 0.20, 0.20 < *δ*_*r*_ ≤ 0.45, and *δ*_*r*_ > 0.45, respectively. Scenarios 2–3 (columns) indicate analysis assuming using the estimated joinpoint, or using the true joinpoint, respectively.

Since Scenario 1 assumes that the population is governed by only one growth rate (e.g. $r_{1}$), bias estimates could not be obtained for $r_{2}$ for this scenario. Regardless, estimates could be determined for Scenarios 2 and 3 (see Fig. 5). Relative bias estimates for $r_{2}$ in Scenario 2 decrease as $\delta_{r}$ increases, but increase as $\delta_{r}$ increases for Scenario 3. The pattern of relative bias for $r_{2}$ matches the pattern of relative bias for $r_{1}$ given Scenario 2, but is opposite of that for Scenario 3. Standard deviations follow a similar pattern (Table 2).

**Table 2:** Mean relative bias of growth rate *r*_2_ (and standard deviation) given *δ*_*r*_ = *r*_1_ − *r*_2_ bins, where *r*_1_ is the growth rate prior to the joinpoint, and *r*_2_ is the growth rate following the joinpoint, by scenario. Scenario 1–3 indicate analysis assuming no joinpoint, using the estimated joinpoint, or using the true joinpoint, respectively.

Bin	Scenario 1	Scenario 2	Scenario 3
*δ*_*r*_ ∈ [0.00,0.20]	0 (NaN)	4.85 (0.45)	1.85 (0.29)
*δ*_*r*_ ∈ (0.20,0.45]	0 (NaN)	−3.14 (0.35)	0.41 (0.10)
*δ*_*r*_ ∈ (0.45,0.70]	0 (NaN)	−0.70 (0.69)	10.08 (0.18)

**Figure 6:**
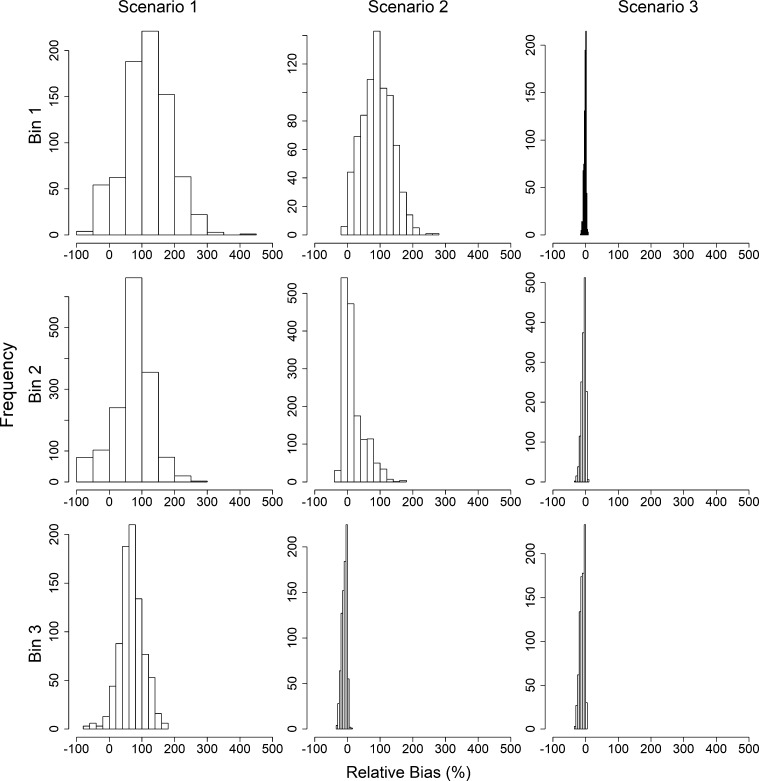
Relative bias (%) of growth rate *q*_1_ given *δ*_*q*_ = *q*_1_ − *q*_2_, where *q*_1_ is the catchability prior to the joinpoint, and *q*_2_ is the catchability following the joinpoint, by scenario. Delta catchability bins 1–3 (rows) include experiments where *δ*_*q*_ ≤ −1.5 × 10^−5^, −1.5 × 10^−5^ < *δ*_*q*_ ≤ −5.0 × 10^−06^, and *δ*_*q*_ > −5.0 × 10^−06^, respectively. Scenario 1–3 (columns) indicate analysis assuming no joinpoint, using the estimated joinpoint, or using the true joinpoint, respectively.

Distributions of the relative bias of the catchability coefficient parameter $q_{1}$ are provided in Fig. 6 for each scenario. For simplicity, the relative bias estimates are summarized given the magnitude of change in catchability before and after the joinpoint and group based on the $\delta_{q}$ (see caption of Fig. 6 for binning).

The mean relative bias for $q_{1}$ is positive for all binning situations when the experiments were analyzed assuming no change to the population dynamics structure. That is, when there is no change in the growth rate or the catchability coefficient (Scenario 1), $q_{1}$ is significantly overestimated. In Scenarios 1 and 2, the magnitude of the relative bias appears to decrease as $\delta_{q}$ increases; in Scenario 3, the magnitude of the relative bias appears to increase as $\delta_{q}$ increases. Further, the relative bias decreases across scenarios within the $\delta_{q}$ bins. That is, for all $\delta_{q}$ bins, the relative bias is smallest when using the true joinpoint. This is followed by the experiments using an estimated join, and finally by the experiments that ignored the joinpoint. For all $\delta_{q}$ bins, the average relative bias was positive, but decreased as $\delta_{q}$ increased. Average relative bias estimates can be found in Table 3

**Table 3:** Mean relative bias of catchability coefficient *q*_1_ (and standard deviation) given *δ*_*q*_ = *q*_1_ − *q*_2_ bins, where *q*_1_ is the catchability coefficient prior to the joinpoint, and *q*_2_ is the catchability coefficient following the joinpoint, by scenario. Scenario 1–3 indicate analysis assuming no joinpoint, using the estimated joinpoint, or using the true joinpoint, respectively.

Bin	Scenario 1	Scenario 2	Scenario 3
*δ*_*q*_ ≤ −1.5 × 10^−5^	116.83 (2.58)	90.69 (1.67)	−1.80 (0.12)
−1.5 × 10^−5^ < *δ*_*q*_ ≤ −5.0 × 10^−06^	72.36 (1.49)	17.97 (0.83)	−6.48 (0.16)
*δ*_*q*_ > −5.0 × 10^−06^	68.06 (1.26)	−9.81 (0.27)	−10.22 (0.25)

As outlined in Table 3, the standard deviations of the mean relative bias decrease as $\delta_{q}$ increases in Scenario 1 and 2, and increases as $\delta_{q}$ increases in Scenario 3.

**Figure 7:**
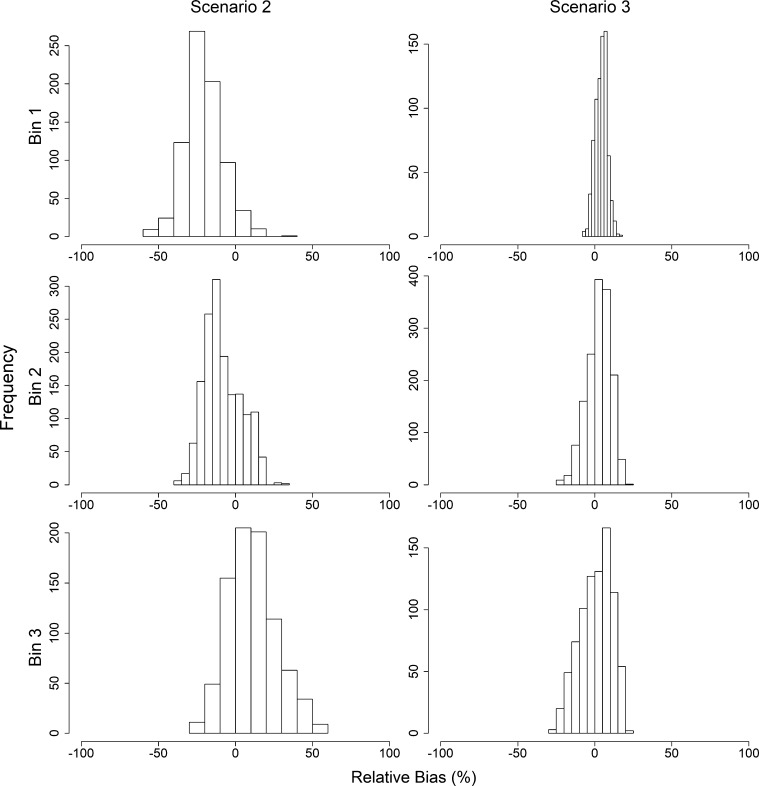
Relative bias (%) of growth rate *q*_2_ given *δ*_*q*_ = *q*_1_ − *q*_2_, where *q*_1_ is the catchability prior to the joinpoint, and *q*_2_ is the catchability following the joinpoint, by scenario. Delta catchability bins 1–3 (rows) include experiments where *δ*_*q*_ ≤ −1.5 × 10^−5^, −1.5 × 10^−5^ < *δ*_*q*_ ≤ −5.0 × 10^−06^, and *δ*_*q*_ > −5.0 × 10^−06^, respectively. Scenario 2–3 (columns) indicate analysis using the estimated joinpoint, or using the true joinpoint, respectively.

Since scenario 1 assumes that the population is governed by only one catchability coefficient (e.g. $q_{1}$), bias estimates could not be determined for $q_{2}$ for this scenario. Regardless, estimates could be determined for Scenarios 2 and 3 (Fig. 7). Relative bias estimates for $q_{2}$ in Scenario 2 increase as $\delta_{q}$ increases, but decrease as $\delta_{q}$ increases for Scenario 3 (Table 4).

**Table 4:** Mean relative bias of catchability coefficient *q*_2_ (and standard deviation) given *δ*_*q*_ = *q*_1_ − *q*_2_ bins, where *q*_1_ is the catchability coefficient prior to the joinpoint, and *q*_2_ is the catchability coefficient following the joinpoint, by scenario. Scenario 1–3 indicate analysis assuming no joinpoint, using the estimated joinpoint, or using the true joinpoint, respectively.

Bin	Scenario 1	Scenario 2	Scenario 3
*δ*_*q*_ ≤ −1.5 × 10^−5^	0 (NaN)	−20.51 (0.44)	4.17 (0.14)
−1.5 × 10^−5^ < *δ*_*q*_ ≤ −5.0 × 10^−06^	0 (NaN)	−8.29 (0.31)	2.63 (0.19)
*δ*_*q*_ > −5.0 × 10^−06^	0 (NaN)	11.12 (0.54)	0.78 (0.35)

## 4 Discussion

The goal of this paper was to present and evaluate the utility of joinpoint regression analysis in the area of ecological risk assessment and population modelling. Joinpoint regression is known to be an effective tool for analyzing changes in trends in epidemiological and cancer-related scenarios; however, little has been done on applying joinpoint regression to ecological risk assessment. To that end, we have proposed that joinpoint regression be combined with surplus production models to allow for the estimation of population parameters that might undergo a sudden change given anthropogenic factors. In the following subsections, we discuss the ability of the joinpoint regression to i) estimate the true joinpoint time $t^{⁎}$, ii) estimate the true joinpoint versus the change in growth rate between periods 1 and 2 (i.e. $\delta_{r}=\left|r_{1}-r_{2}\right|$), iii) estimate the true joinpoint versus the change in catchability coefficients between periods 1 and 2 (i.e. $\delta_{q}=\left|q_{1}-q_{2}\right|$), iv) identify the length of the transition period during which a stable population appeared following the joinpoint (i.e. $\mathrm{abs}\left(t_{1}^{⁎}-t_{2}^{⁎}\right)$) and, v) improve the estimates of growth rate and catchability compared to standard methods (i.e. when joinpoints are ignored), or when the joinpoint is known *a priori*.

### 4.1 Estimation of true joinpoint time

The joinpoint regression model was able to effectively estimate the true joinpoint time $t_{1}^{⁎}$. That is, given a simulated time series that included a point in time where the population dynamics changed, the model was able to provide a reasonable estimate of when this change occured. Although interval estimation was beyond the scope of the work for this study, Muggeo (2017) provides a smoothed score-based approach for interval estimation for joinpoint regression. This interval estimation method, now included in newer versions of the *segmented* packaged in R, should be considered for future uses of this model when providing estimates of true joinpoint time (Muggeo, 2017).

It is important to note, however, that joinpoint regression models tended to perform better (i.e. estimate population parameters with low absolute relative bias) when the magnitude of change in the growth rate was *large*. This is evident based on the variances observed for each level of $\delta_{r}$ in the simulations. The variances observed were approximately 5 times higher when $\delta_{r}<0.10$ compared to when $\delta_{r}\geq 0.10$. This can be viewed as a double-edged sword. On one hand, the joinpoint regression model may be able to prove itself effective to be able to detect changes in population trends that are not obvious by inspection alone, allowing managers to potentially take action sooner if there is a negative change in population dynamics. On the other hand, joinpoint regression could be viewed as too sensitive of a model and estimate a change in population dynamics when there is no change. Further studies should investigate the effectiveness of joinpoint regression modelling in populations with little to no change in population dynamics.

### 4.2 Transition period length

Transition period length was a metric used to estimate how long it took for a population to re-stabilize after a change in population dynamics. This was accomplished by allowing the joinpoint regression model to estimate two joinpoints: $t_{1}^{⁎}$ —when the population dynamics changed, and $t_{2}^{⁎}$ —the point in time where the population was re-stabilized following the change in population dynamics. The transition period was calculated as the average of the difference in these two estimated joinpoints.

On average, the transition period length was approximately 7 years. However, this varied heavily depending on the magnitude of change in growth rate and catchability. For instance, in cases where the magnitude of change in population dynamics was small, the transition length tended to be longer. Two possible reasons exist to explain this. One explanation could be that when there was a small change in population dynamics, the overall population may have only experienced a gentle increase or decrease in total population over a long period of time before re-stabilizing (that is, neither increasing or decreasing in population on average), thus drawing out the transition period time. Another explanation could be that when there was a small change in population dynamics, the joinpoint regression model wasn't sensitive enough to pick up a distinct time when the population re-stabilized, which could also draw out the transition period. Future studies should investigate the effectiveness of joinpoint regression to provide an accurate transition period length in populations where the dynamics only change slightly.

### 4.3 Estimates of population dynamics

Simulated time series data were analyzed to estimate the growth rate and catchability coefficient for each of the three scenarios. As mentioned, since scenario 1 did not assume any change in population dynamics, only one growth rate and catchability coefficient were calculated for the entire time series. This contrasts with scenario 2 and scenario 3 which had a growth rate and catchability coefficient estimated for the period before the joinpoint and the period after the joinpoint.

For each scenario, mean relative bias was used as an indicator of how close the estimates were to the true growth rate or catchability coefficient. Compared to simulations assuming no change in population dynamics, the use of either an estimated joinpoint or the true joinpoint appeared to significantly decrease the mean relative bias of population parameter estimates. That is, when population parameters suddenly change, population dynamics could be more accurately estimated when using joinpoint regression. In comparing mean relative bias between estimates obtained with an estimated joinpoint versus the true joinpoint, while there are some minor differences, they were still significantly less biased than the scenario assuming no joinpoint. This would imply that even if a joinpoint is not known *a priori*, the use of an estimated joinpoint is still sufficient for more accurate estimates of population parameters.

### 4.4 Limitations and future work

In this study, we only investigated the case of a single joinpoint in time. Further studies could explore the effectiveness of joinpoint regression parameter estimates with models containing two or more joinpoints in time. With these multiple joinpoints, edge properties can be explored by having a joinpoint close to the beginning of a simulation, close to the end of a simulation, or both.

All of the scenarios we investigated used a constant carrying capacity *K* throughout the simulation. This constant carrying capacity added an overall stability to the population once it stabilized after the preliminary 32 year period. Future studies should investigate joinpoint regression's efficiency in a simulation that may have either a change in *K* at a certain point in time or a varying *K* over a long period of time.

This study only investigated the case of a varying growth rate or catchability coefficient, where the population parameters before and after the joinpoint were sampled from finite and discrete sets. Further studies could allow *r* or *q* to be sampled from a distribution, rather than randomly sampled from a finite set.

It's worth noting that, as mentioned before, growth rate *r* is typically a stable population parameter (Hilborn and Walters, 2001), however for the purposes of this study, we chose to have a change in growth rate at time *t* to investigate whether this change could even be picked up by the joinpoint model. Additionally, in a typical fishery environment, the growth rate *r*, if it does change, generally increases with increased exploitation. In our study, however, we chose to simply investigate a change in growth rate *per se*, and not necessarily related to increased exploitation. Future studies could seek to investigate a change in growth rate as it pertains to a change in exploitation.

All of the scenarios we investigated assumed nothing about the gear used for harvesting, except that it is constant through time. Future studies could look at whether a joinpoint regression can pick up changes in gear used for harvesting, regardless if there was an actual change in population parameters.

## 5 Conclusions

The results shown here are preliminary results for determining the effectiveness of joinpoint regression to improve population parameter estimates from standard surplus production models. In this paper, we aimed to 1) determine if joinpoint regression can be used to identify if and when population structure changes occur based on time series of abundance, and 2) compare and contrast population parameter estimates derived through joinpoint and surplus production methods to those derived from standard surplus production methods alone.

The joinpoint regression was consistently able to estimate the true joinpoint $t^{⁎}$, although these estimates tended to be more accurate as the magnitude of change in population parameters increased. This was obvious in observing the population parameters estimates of both growth rate and catchability coefficient before and after the joinpoint in that the variance of estimates tended to decrease as the magnitude of change in either of the parameters increased. The change of magnitude in these population parameters also played a factor in calculating transition period length in that a smaller change of magnitude tended to correspond with a longer transition period. Finally, relative bias of population parameter estimates tended to decrease when using either an estimated joinpoint time or true joinpoint time compared to estimates where no joinpoint time was assumed; that is, when using the joinpoint, whether it was the estimated or the true joinpoint, the estimates in population parameters were more accurate to the true value of the population parameter compared to the estimates that assume no joinpoint.

We have shown here how joinpoint regression can be used to improve population parameter estimation in surplus production models. However, the use of joinpoint regression should be considered for other types of risk assessment modelling, such as age- or stage-structured population modelling, to gain a more accurate and complete understanding of population dynamics and changes that may occur through time.

## Declarations

### Author contribution statement

Daniel Gillis: Conceived and designed the experiments; Performed the experiments; Analyzed and interpreted the data; Contributed reagents, materials, analysis tools or data; Wrote the paper.

Brandon P.M. Edwards: Performed the experiments; Analyzed and interpreted the data; Contributed reagents, materials, analysis tools or data; Wrote the paper.

### Funding statement

This research did not receive any specific grant from funding agencies in the public, commercial, or not-for-profit sectors.

### Competing interest statement

The authors declare no conflict of interest.

### Additional information

Supplementary content related to this article has been published online at 10.1016/j.heliyon.2019.e02515.

## Appendix A Supplementary material

JP SUPPLEMENTARY 1 - JAGS CODE_V2.ZIP JAGS Code. JP SUPPLEMENTARY 1 - JAGS CODE_V2.ZIP

JP SUPPLEMENTARY 2 - R CODE_V2.ZIP R Code. JP SUPPLEMENTARY 2 - R CODE_V2.ZIP
